# Developing High-Performance and Low-Cost Paint Thermoelectric Materials for Low-Midtemperature Applications

**DOI:** 10.1021/acsami.3c19309

**Published:** 2024-03-01

**Authors:** Muhammed Yilmaz, Aminu Yusuf, Koray Gurkan, Sedat Ballikaya

**Affiliations:** † Department of Chemical Engineering, Istanbul University-Cerrahpaşa, Avcılar 34320, Istanbul, Turkey; ‡ Department of Engineering Sciences, Istanbul University-Cerrahpaşa, Avcılar 34320, Istanbul, Turkey; § Department of Electrical and Electronics Engineering, Istanbul University-Cerrahpaşa, Avcılar 34320, Istanbul, Turkey

**Keywords:** low-cost, nontoxic, paint thermoelectric, paper thermoelectric generator, scalable, wearable

## Abstract

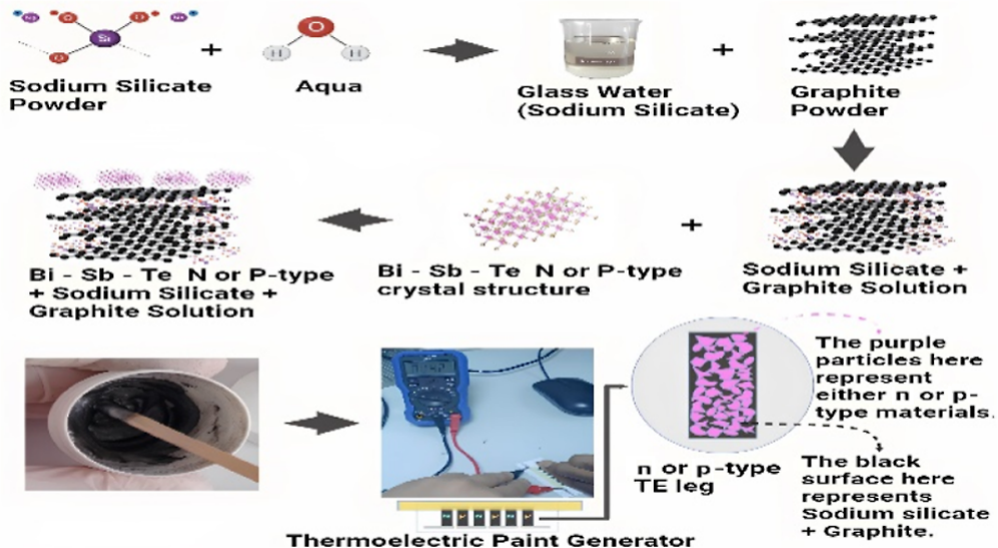

Most thermoelectric (TE) materials used to convert heat energy into electrical energy are expensive and, to a certain degree, toxic. Moreover, due to the chemical complexity in the synthesis process, some of the TE materials are not reproducible. Similarly, the scarcity of TE materials hampers their scalability. To address the above issues, this study presents an inexpensive, nontoxic, scalable, and highly reproducible paint-based TE module for the conversion of heat energy to electrical energy. Transport properties with structural analysis indicate that the electrical conductivity of the paint TE material is controlled by the concentration of graphite and sodium silicate, while the Seebeck coefficient is dominated by the ratio of n- and p-type Bi–Sb–Te. The results indicate that the as-developed TE module can withstand an operating temperature of up to 160 °C. At a temperature of 57 °C, the highest power factors of the as-synthesized n- and p-type TE paints are 1.34 and 1.42 μW/(cm K^2^), respectively. It is also found that the TE module can have a higher output voltage when the cold side of the TE module is allowed to float in the air in comparison to that when it is in contact with the human body. The performance of the paint-based TE module is measured on five parts of the body, namely, the chest, palm, leg, wrist, and neck; the wrist has the highest open-circuit voltage of 1.9 mV, indicating its suitability for wearable applications. Finally, at a temperature gradient of 30 °C, a maximum output power of 6.8 μW is attained.

## 1 Introduction

Most of the global energy sources dissipate waste heat energy to the surroundings; this waste heat energy if utilized efficiently can alleviate the energy crisis the world is currently facing. The waste heat energy can be used by steam turbines and thermoelectric generators (TEGs) as well as in domestic and industrial heating applications. The choice of how the waste heat is utilized depends on the primary energy source. For example, the human body dissipates a significant amount of heat to the surroundings. Typically, an adult human body can dissipate heat energy in the range of 60–180 W.^1^ Suppose that the total adult population in the world is 3 billion, and a device with 5% electrical energy conversion efficiency is employed to utilize the heat dissipated by each adult, a total annual energy of 78.84 TW h would be generated. A TEG is suitable for this application because it converts heat energy directly into electrical energy.^2−6^ TEGs can operate day and night with zero maintenance and no carbon emissions.^7^ Conversion of heat energy into electrical energy strongly depends on the value of the figure of merit (*z*) of the thermoelectric (TE) material; in a dimensionless form, it is given as *zT* = *S*^2^σ*T*/*k*,^8,9^ where *S* is the Seebeck coefficient, σ is the electrical conductivity, *T* is the absolute temperature, and *k* is the total thermal conductivity.

To harvest heat energy efficiently from curved-heat sources, flexible TEGs should be used. Flexible TEGs can be either in a cross-plane configuration, where the TE legs are perpendicular to the substrate, or in an in-plane configuration, where the TE legs lie in the same plane with the substrate. Usually, cross-plane TEGs consist of bulk TE materials, which are rigid and brittle. In that case, flexible substrates are needed to achieve flexibility.^7,10−13^ Other ways of achieving flexibility are by creating voids in the substrate^14^ as well as using stretchable liquid-metal interconnects.^15^ On the other hand, the in-plane TEGs are characterized by high flexibility and mechanical stability,^16,17^ and the production process is both reproducible and scalable.^18^ An in-plane TEG fabricated using state-of-the-art techniques can be competitive with the cross-plane TEGs.^19^ Furthermore, it has been shown that optimizing the structure of the TE legs from a single layer to an annular structure enhances the output power of the flexible TEG by 610%; this is in addition to saving a lot of space and being suitable for versatile applications.^20^

Paper-based flexible TEGs are increasingly attracting attention because they can be fabricated easily and are inexpensive and lightweight.^21,22^ Generally, in paper-based TEGs, TE legs are brush-painted on the surface of a paper substrate. Paint-based TEGs are made up of organic/inorganic TE materials that are either solvent-free or mixed with a solvent to form TE paint. In a solvent-free TE paint, HB pencils (graphite) are used to draw the n- and p-type TE legs on substrates, usually paper. Here, p-type graphite TE legs can be transformed into an n-type TE leg by dipping it into the solution of polyethylenimine (PEI) and allowing it to dry.^23^ Since the graphite traces have high electrical conductivity and very low Seebeck coefficient,^24,25^ the power factor of the TEG is low. On the other hand, organic TE paints (mixed with solvents) can have a higher performance due to their higher Seebeck coefficient and lower thermal conductivity in comparison with that of the graphite trace TE paints.^26^ Preparation of organic TE materials requires complex material engineering process,^27−30^ and these materials are generally characterized by high electrical resistivity; this pushes researchers to look for other suitable TE materials (paints). Recently, it has been shown that p-type Na_1.4_Co_2_O_4_ and n-type Na_*x*_Co_2_O_4_ synthesized using the self-flux method can be applied to a print paper to form a flexible TEG; the module generated a maximum power factor of 223 μW m^–1^ K^–2^.^31^

Other studies explored the use of inorganic TE materials as TE paints;^32−35^ this is because of their superior performance in comparison with that of organic TE paints. Generally, inorganic TE materials are synthesized and then mixed with a solvent to form the TE paint.^36^ The geometry of the paint-based TE legs and the interconnect can influence the output performance of the TEGs.^32^ Substrates with good electrical conductivity and durability should be chosen to enable the TE legs of paint-based TEGs to generate electricity. For example, when paint-based TEGs on standard paper and polyester paper substrates are examined, the output power of the module with polyester paper substrate is over three times higher than that of the module with standard paper substrate.^33^ It is often difficult to create a temperature gradient across the hot and cold sides of paint-based TEGs when considered for wearable applications; this is because both sides lie in the same plane. Without a reasonable temperature gradient, electrical energy cannot be created. Other factors that hinder the commercialization of TEGs include the high cost of the materials, toxicity, scarcity, and difficulty in the material synthesis.

Having said that, this study is carried out to address the above issues by presenting an inexpensive, nontoxic, scalable, and highly reproducible paint-based TE module for converting heat energy into electrical energy. The contributions include the synthesis of p- and n-type inorganic TE materials, determination of the optimum concentration of inexpensive and nontoxic graphite powder and sodium silicate solvent that should be added to the inorganic TE materials, development of a paper-based TEG, and exploration of the most suitable position on the human body for wearable applications. Finally, the study suggests a way to enhance the performance of the paper-based TEG by allowing the cold side to float in the air.

## 2 Materials and Methods

The chemical elements Bi 99.999%, Sb 99.999%, Se 99.999%, Te 99.999%, and Cu 99.98% (Sigma-Aldrich) were mixed according to 2% Cu–Bi_2_Te_2.7_Se_0.3_ and Sb_1.5_Bi_0.5_Te_3_ stoichiometric ratios in a glovebox and then loaded into zirconium oxide containers; zirconium oxide balls of weight ten times the weight of each sample were added into zirconium oxide containers. This ratio was chosen to ensure homogeneity in the fine powder during the 8 h, 600 rpm ball milling process (Optosense). Graphite powder (CAS no: 7782-42-5) weighing 16.5% of the TE material (2% Cu–Bi_2_Te_2.7_Se_0.3_/Sb_1.5_Bi_0.5_Te_3_) was mixed with the TE material in a bowl for 1 min. Thereafter, 0.13 mL of Na_2_O(SiO_2_)_*x*_·*x*H_2_O (CAS no: 9003-04-7) was added into the bowl, and the mixture was mixed for 5 min to achieve a homogeneous solution. The graphite, being nontoxic and cheaper than the inorganic TE material, was added to increase the quantity and to improve the electrical conductivity of the TE material. Sodium silicate is also a cheap and nontoxic inorganic solvent; it provides a good synergy between graphite and TE materials. Furthermore, its adhesive property makes the solution ideally suitable for applications to many surfaces. Thus, the electrical conductivity can be controlled by the amounts of graphite and sodium silicate, whereas the Seebeck coefficient can be controlled by the n- and p-type Bi–Sb–Te additive ratio.

In view of the above, the as-synthesized TE paint is cheap, nontoxic, scalable, and highly reproducible. These features offset most of the drawbacks of conventional TE materials.

**Figure 1:**
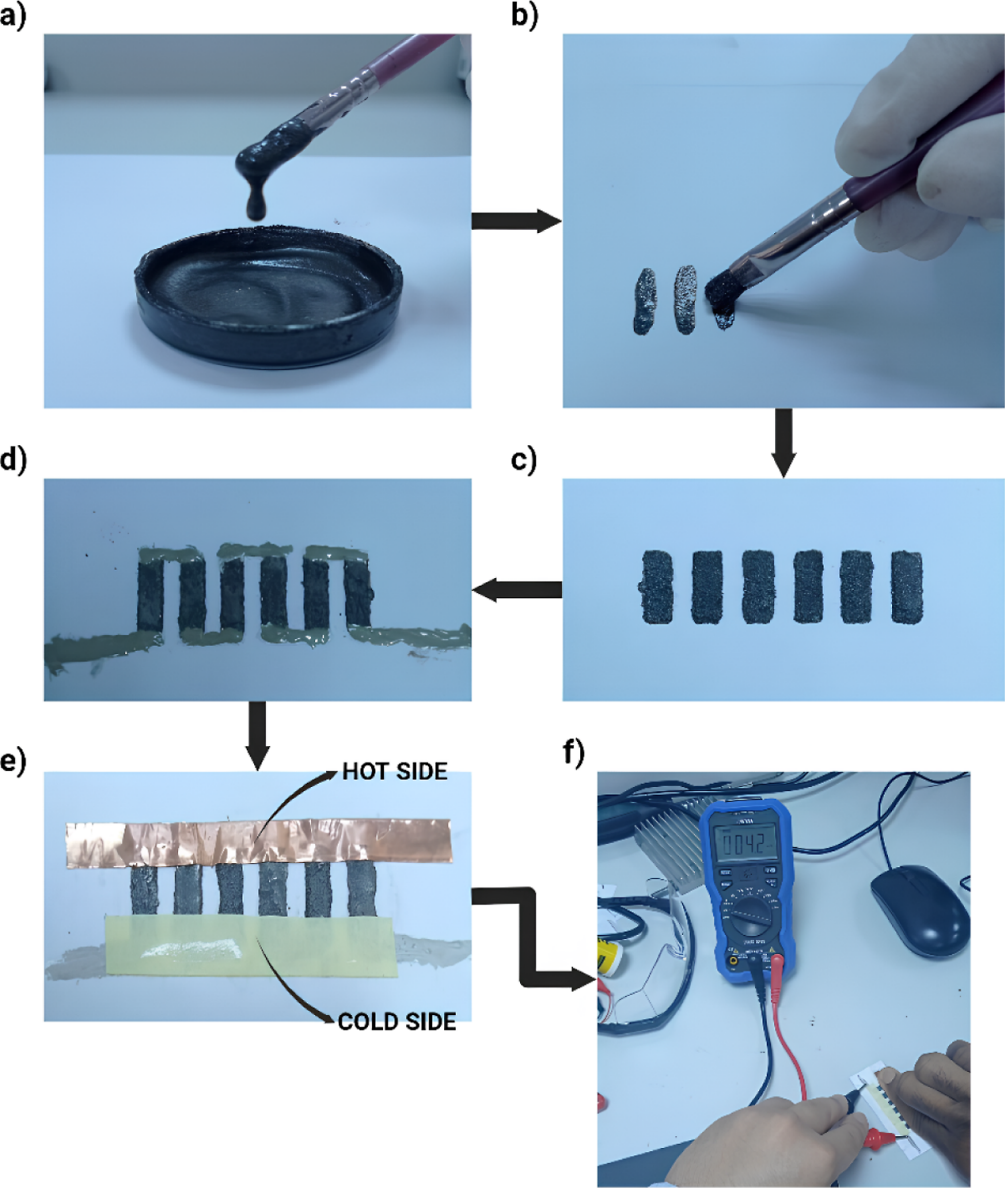
Paint-based TEG: (a) paint-based TE material, (b) brush painting the TE legs, (c) painted TE legs on a paper substrate, (d) interconnecting the painted legs with silver paste, (e) creating hot and cold sides using double-sided tape, and (f) converting body heat to electrical energy.

The as-synthesized solution (TE paint) was applied to a standard office paper in the form of TE legs and allowed to dry at room temperature (25 °C) for 30 min. The drying period can be reduced to 5 min simply by placing the paper on a hot plate at 50 °C. Subsequently, each TE leg was gently compacted on the paper to reduce the gaps between the graphite, the TE material, and the paper. Two TE legs (n- and p-type) were used for Seebeck and electrical resistivity measurement (ZEM-3 Ulvac Co.). Hall effect measurements were performed at room temperature (DX-50 Hall System from the Xiamen Dexing Magnet). Likewise, a small quantity of the dried solution was also used for powder X-ray diffraction (PXRD) analysis (Rigaku Ultima X-ray diffractometer) and scanning electron microscopy (SEM) analysis (Phenon ProX Scanning Electron Microscope). The paste was used to connect the TE legs; this ensures flexibility and eliminates excessive contact resistance that would have been present if metal electrodes were used. In order to achieve a large temperature gradient, a copper plate was placed on the hot side, while paper tape was used on the cold side of the TE legs. If the paint-based TEG is to be worn, the presence of copper enhances heat transfer on the hot side, while the paper tape reduces heat absorption on the cold side. If, however, the cold side is not in contact with the heat source, then there would be no need for the copper plate and the paper tape. The development process of the paint-based paper TEG is shown in Figure 1a–e, while Figure 1f shows a typical measurement setup for the paint-based paper TEG.

**Figure 2:**
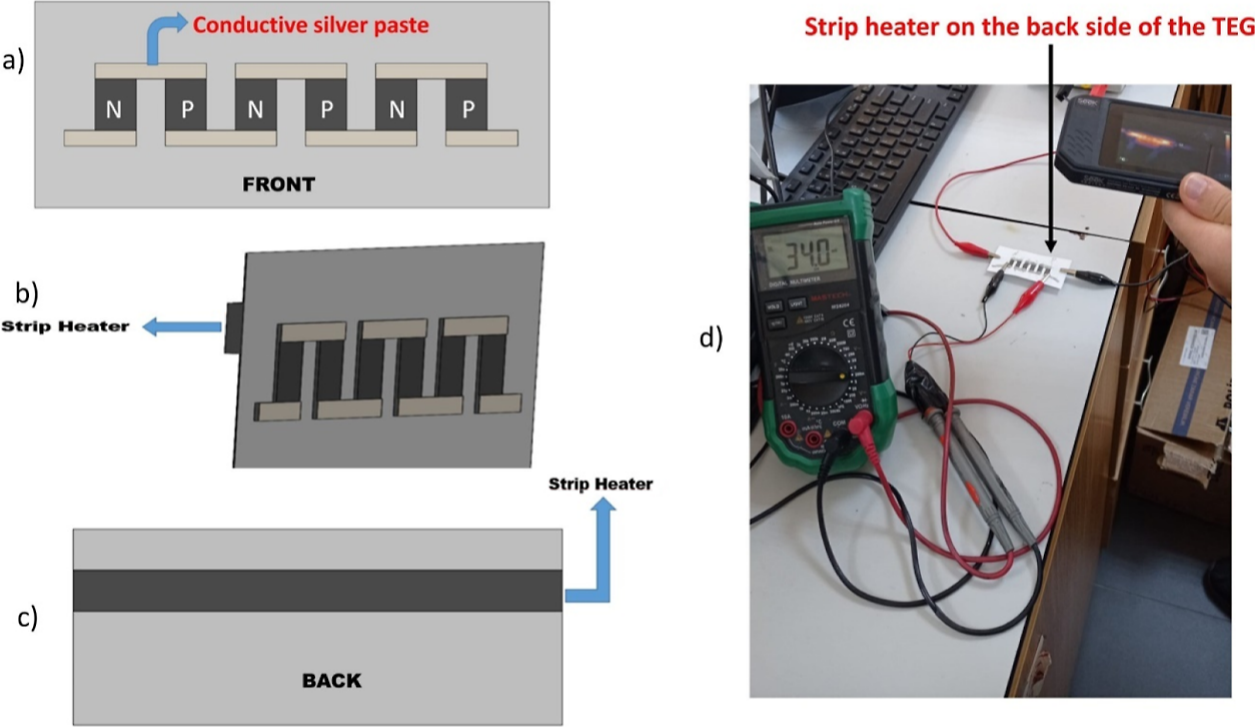
Paint-based TEG: (a) front view, (b) lateral view, (c) back view, and (d) measurement of open-circuit voltage.

A 2D sketch of the paint-based TEG incorporated with a strip heater on the back side is shown in Figure 2a–c, while Figure 2d presents the measurement of an open-circuit voltage.

The open-circuit voltage of the TEG is given as
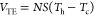
1

while the electrical current passing through the device is given as
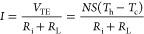
2where *N* is the number of thermocouples, *S* is the average Seebeck coefficient, *T*_h_ is the hot side temperature, *T*_c_ is the cold side temperature, *R*_i_ is the internal resistance, and *R*_L_ is the load resistance. The output power of the TEG is computed as follows
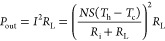
3

According to the maximum power transfer, the maximum power is achieved when load resistance is equal to the internal resistance as given by
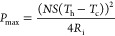
4

## 3 Results and Discussion

### 3.1 Characterization of the Thermoelectric Paint

Characterization of the TE paint was carried out to determine its suitability for TE applications. At first, the optimum concentration of sodium–silicate with graphite was determined, and then n- and p-type Bi–Sb–Te compounds were added to the solution to form TE based-paint. The optimum electrical conductivity was determined with the optimization of the sodium silicate and graphite concentration. To understand the physical relationship between the graphite and the sodium silicate, hall coefficient and electrical conductivity measurements were performed as shown in Table 1.

**Table 1:** Hall Measurements of Different Concentrations of Graphite and Sodium Silicate

material concentration [graphite (G)/sodium silicate (SS)]	Hall coefficient (cm^3^/C)	carrier concentration (1/cm^3^)	carrier mobility [cm^2^/(V s)]	electrical conductivity (S/cm)
1g_G + 1.1mL_SS	–0.2889011	1.30 × 10^17^	3.195854	24.90767
1g_G + 0.9mL_SS	–0.1632004	1.53 × 10^17^	4.737867	29.03098
1g_G + 0.8mL_SS	–0.121425	3.09 × 10^17^	5.442208	44.81949
1g_G + 0.7mL_SS	–0.152275	4.10 × 10^16^	11.09237	72.84431

As can be seen in Table 1, both carrier mobility and electrical conductivity increase as the sodium silicate decreases (the graphite content increases). This is important as it shows that graphite greatly contributes to the determination of the electrical conductivity of the paint. When the sodium silicate is reduced below 0.7 mL, the viscosity of the solution (graphite plus sodium silicate) decreases, thus increasing its fluidity. Thus, it was found that the graphite ratio in the solution should not be more than 70%. It should be noted that the ratio of sodium silicate and graphite is changed after adding n-type and p-type TE materials to preserve the viscosity and obtain a high Seebeck coefficient and electrical conductivity, as shown in Table 2. As seen in Table 2, the optimum concentration of TE paint was obtained with 0.071 g of graphite powder, 0.13 mL of sodium silicate, and 0.429 g of n-/p-type TE compound.

**Table 2:** Optimization of the Concentration of Sodium Silicate, Graphite, and n- and p-Type TE Materials at the Measurement Temperature of 60 °C

graphite (g)	sodium silicate (mL)	n-type TE compound (g)	electrical cond. (S/cm)	Seebeck coeff. (μV/K)	power factor [μW/(cm K^2^)]
0.2	0.2	0.3	78.6	–17	0.023
0.1	0.15	0.4	72.3	–30	0.065
0.082	0.14	0.417	63.4	–112	0.80
0.071	0.13	0.429	60	–147	1.30
0.062	0.12	0.438	48.4	–92	0.41

graphite (g)	sodium silicate (mL)	p-type TE compound (g)	electrical cond. (S/cm)	Seebeck coeff. (μV/K)	power factor [μW/(cm K^2^)]
0.2	0.2	0.3	84	22	0.04
0.1	0.15	0.4	73	38	0.105
0.082	0.14	0.417	66	116	0.88
0.071	0.13	0.429	58	146	1.23
0.062	0.12	0.438	42	84	0.3

**Figure 3:**
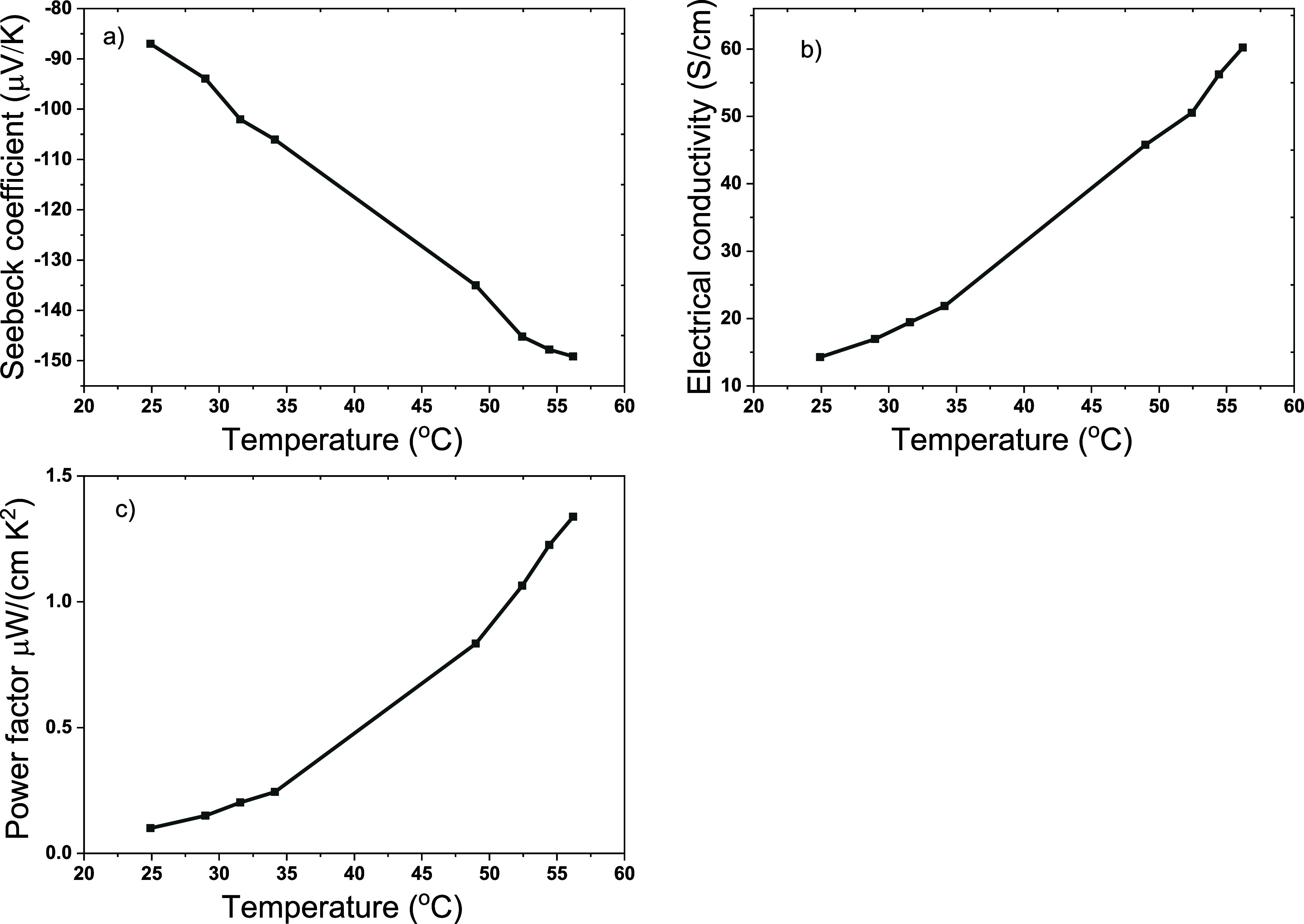
Transport properties of the n-type paint-based TE material: (a) Seebeck coefficient, (b) electrical conductivity, and (c) power factor.

A maximum temperature of 57 °C is considered during the measurement because the module is designed for wearable applications in which the temperature of operation is less than 57 °C. Figure 3a presents the variation of the Seebeck coefficient of the n-type TE paint with temperature. The Seebeck coefficient monotonically increases with an increase in temperature; for example, the Seebeck coefficient is −86 μV/K at room temperature and increases to −150 μV/K at 57 °C. In Figure 3b, the electrical conductivity also increases with an increase in the temperature. A maximum electrical conductivity of 63 S/cm is obtained at a maximum temperature of 57 °C. Figure 3c presents the power factor of the n-type TE paint and is derived from the product of the square of the Seebeck coefficient and the electrical conductivity (*S*^2^σ). It can be seen that the power factor increases with the increase in temperature, because both the Seebeck coefficient and the electrical conductivity increase with the temperature. From room temperature to 57 °C, the power factor increases from 0.1 to 1.34 μW/(cm K^2^). This result indicates that the n-type TE paint can have a higher performance at higher temperatures.

**Figure 4:**
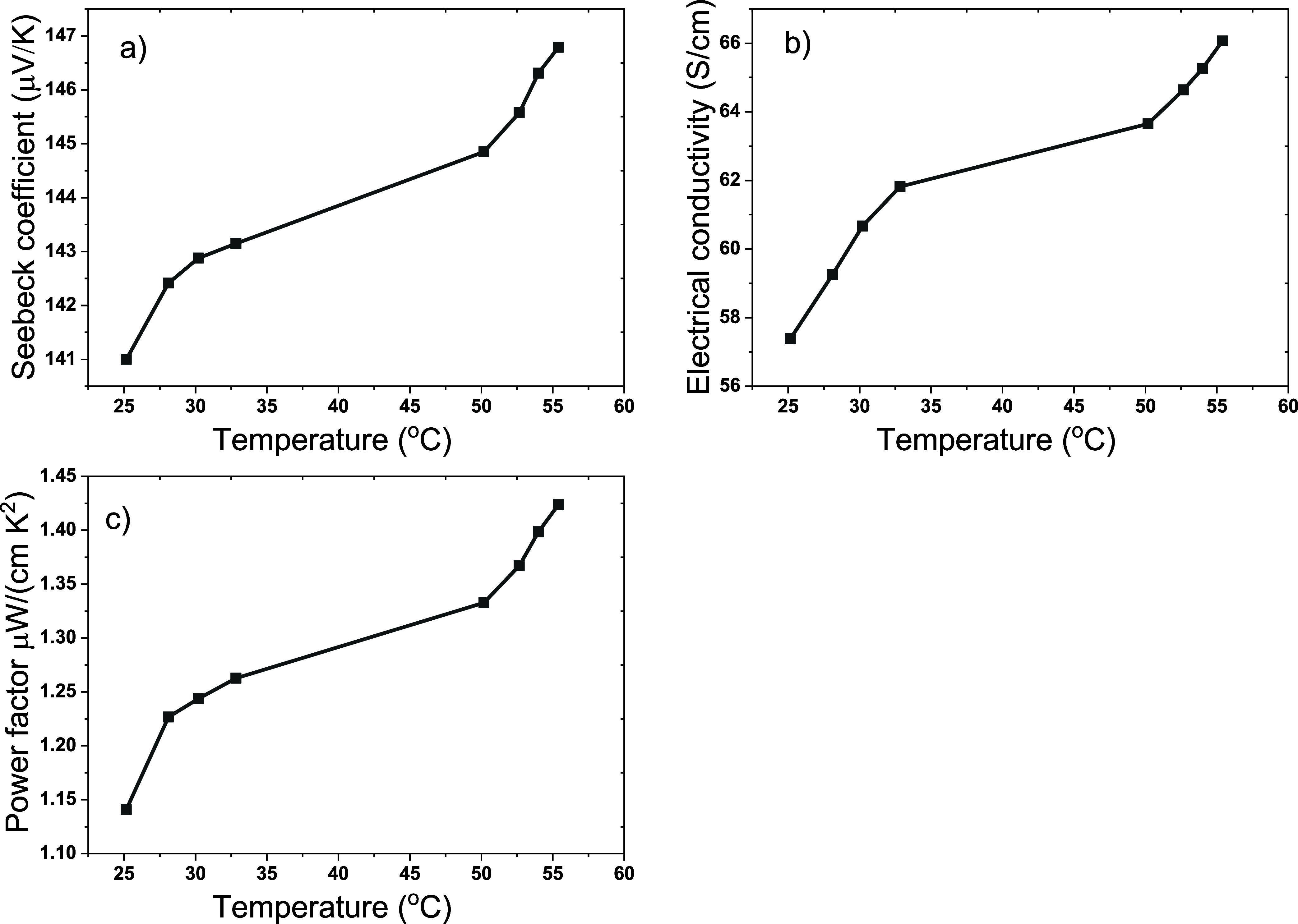
Transport properties of the p-type paint-based TE material: (a) Seebeck coefficient, (b) electrical conductivity, and (c) power factor.

Figure 4a shows the variation of the Seebeck coefficient of the p-type TE paint with temperature. As the temperature increases from 25 to 57 °C, the Seebeck coefficient increases from 141 to 147 μV/K. The electrical conductivity of the p-type TE paint increases gradually with the increase in the temperature as shown in Figure 4b. At the maximum temperature of 57 °C, the highest electrical conductivity of 66.4 S/cm is obtained; this value is slightly higher than that of the n-type TE paint obtained at the same temperature. The power factor increases with the increase in temperature due to the increase of the two transport properties, as shown in Figure 4c. The highest power factor of 1.43 μW/(cm K^2^) is obtained at the highest temperature of 57 °C. The power factor is higher than that of the n-type TE paint because the overall product of the transport properties is higher in the former. These results corroborate the already-known fact that the performance of p-type TE material is generally higher than that of the n-type TE material.^37^

**Figure 5:**
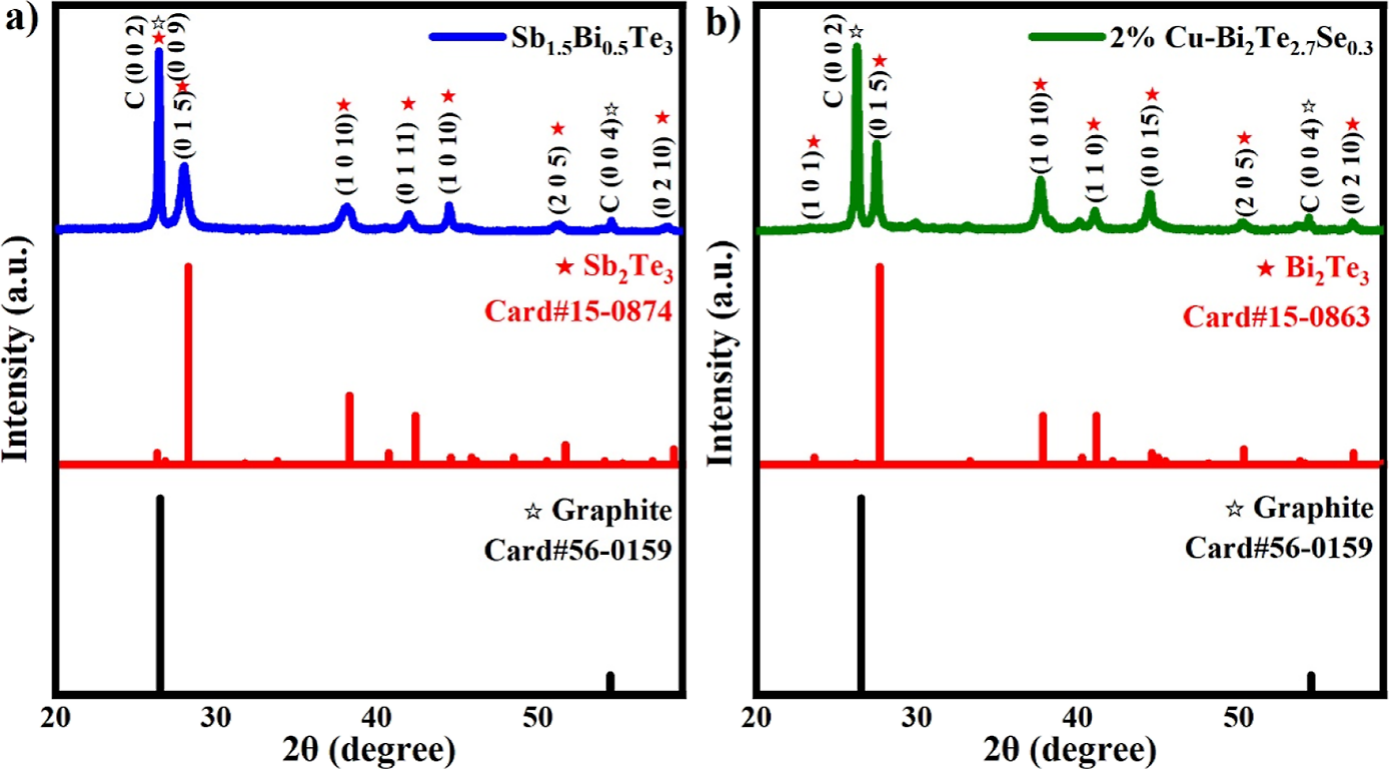
PXRD graphs: (a) p-type Sb_1.5_Bi_0.5_Te_3_ and (b) n-type 2% Cu–Bi_2_Te_2.7_Se_0.3_.

**Figure 6:**
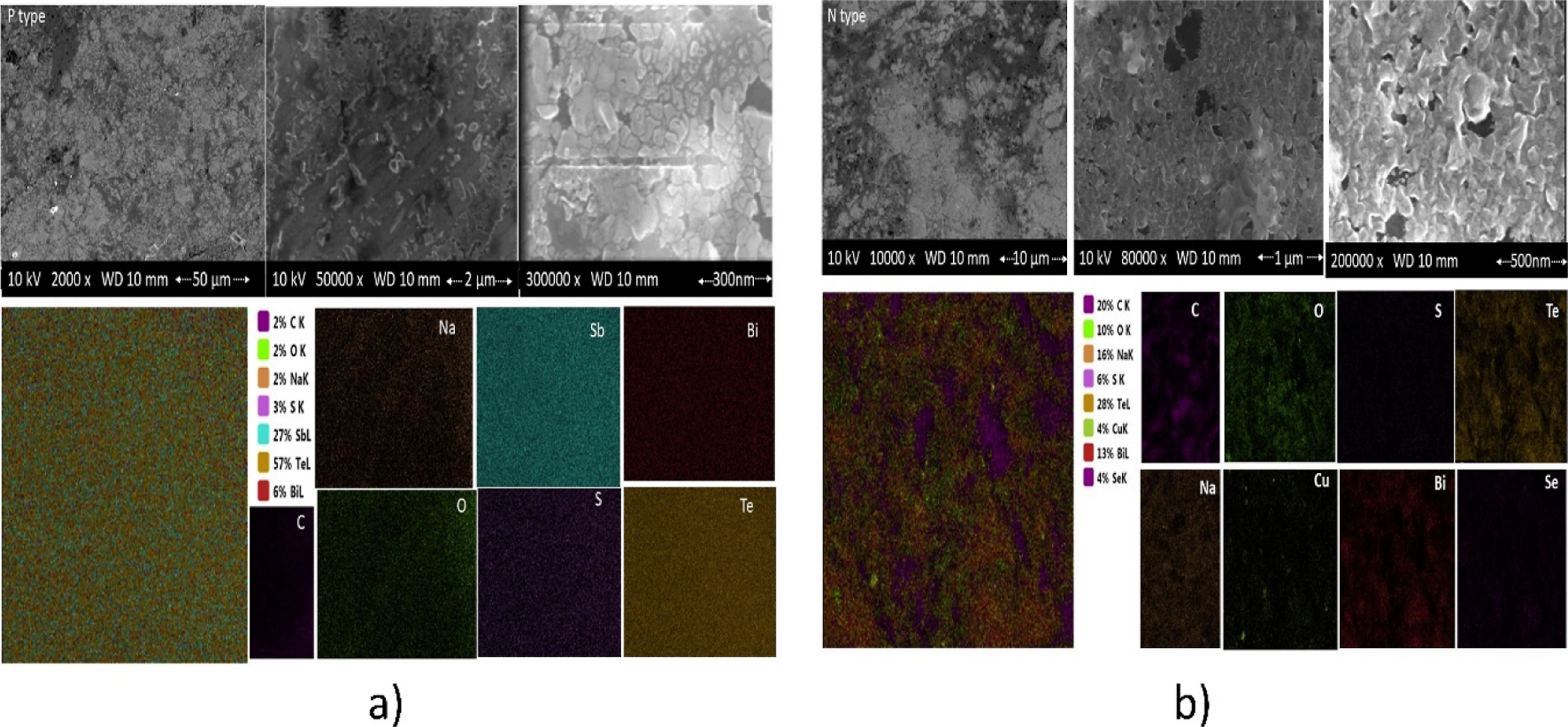
SEM analysis: (a) p-type Sb_1.5_Bi_0.5_Te_3_ and (b) n-type 2% Cu–Bi_2_Te_2.7_Se_0.3_.

The temperature dependence of the Seebeck coefficient and electrical conductivity tends to exhibit opposite trends. In other words, while electrical conductivity tends to increase with temperature, the Seebeck coefficient shows a tendency to decrease or vice versa. However, as seen from the transport data provided in Figures 3 and 4, both n-type and p-type compounds demonstrate an increase in both the Seebeck coefficient and electrical conductivity with temperature. The reason for this unusual situation can be explained as follows. As shown in Table 1, which illustrates the impact of sodium silicate-graphite ratios on electrical conductivity, mobility, and carrier density, it is observed that the graphite ratio plays a significant role in the increase in electrical conductivity, while the amount of Bi–Sb–Te dominates the increase in the Seebeck coefficient (see in Table 2). The SEM images along with PXRD results (Figures 5 and 6) provide supporting evidence for this. In particular, SEM–EDX images show that graphite spreads on the substrate surface due to the influence of sodium silicate, while the Bi–Sb–Te compounds forms clusters in the structure. Therefore, the increase in electrical conductivity can be associated with the semiconducting behavior of graphite. In other words, with the increase in temperature, the thermally induced carrier density originating from graphite may have increased. On the other hand, the increase in the Seebeck coefficient can be related to the scattering or trapping of low-energy carriers within the increased charged carrier density at the cluster boundaries formed by Bi–Sb–Te. Although a definitive interpretation could be provided with detailed TEM (transmission electron microscopy) and temperature-dependent Hall measurement results, the occurrence of this phenomenon in both n-type and p-type compounds is supported by the results in Tables 1 and 2 as well as the microstructural characteristics (see Figures 5 and 6).^2,38^

To further understand the relationship between the sodium silicate–graphite and TE compounds, the microstructural properties of the TE paints were characterized by PXRD and SEM analyses. Figure 5a,b shows the PXRD pattern obtained for the and p- and n-type TE paints at room temperature.

As observed from the PXRD graphs, carbon (Card# 56-015) is prominently present in both n-type and p-type structures around 27°. In addition to the graphite introduced into the structure, water-based sodium silicate may have facilitated the spreading of graphite on the paper surface by increasing its surface area. Peaks other than carbon in both materials are consistent with the literature; the main peaks of Figure 5a reflect the natural crystal structures of Sb_2_Te_3_ (Card# 15-0874), while Figure 5b reflects the natural crystal structures of Bi_2_Te_3_ (Card# 15-0863).

Figure 6a,b presents the scanning electron microscopy–energy-dispersive X-ray (SEM–EDX) analysis of the p- and n-type TE materials, respectively. These figures indicate that both samples generally have identical stoichiometric compositions. However, as seen in the PXRD pattern, the carbon amount in the n-type TE paint seems to be higher than the amount present in the p-type TE paint. The inhomogeneous distribution of carbon can be seen in color mapping. The SEM patterns show that all samples have an island-shaped distribution of grains.

Sodium silicate–graphite combination is generally used as a bipolar plate for proton exchange membrane in fuel cells. Sodium silicate changes the surface energy of graphite, increases its polarity, and breaks the van der Waals forces of the layer, thus not only enhancing the electrical conductivity but also expanding the surface area of graphite. As seen in Figure 6, SEM images and the color map demonstrate that the TE coating consists of island-like structures. The islands predominantly comprise n- and p-compounds, while the connecting structure (resembling a sea) is composed of a graphite-based material. Although the accuracy of this interpretation can be further confirmed by a detailed microstructural analysis such as TEM analysis, the SEM and PXRD results indicate the potential validity of this approach.

### 3.2 Open-Circuit Voltage of the Paint-Based TEG

**Figure 7:**
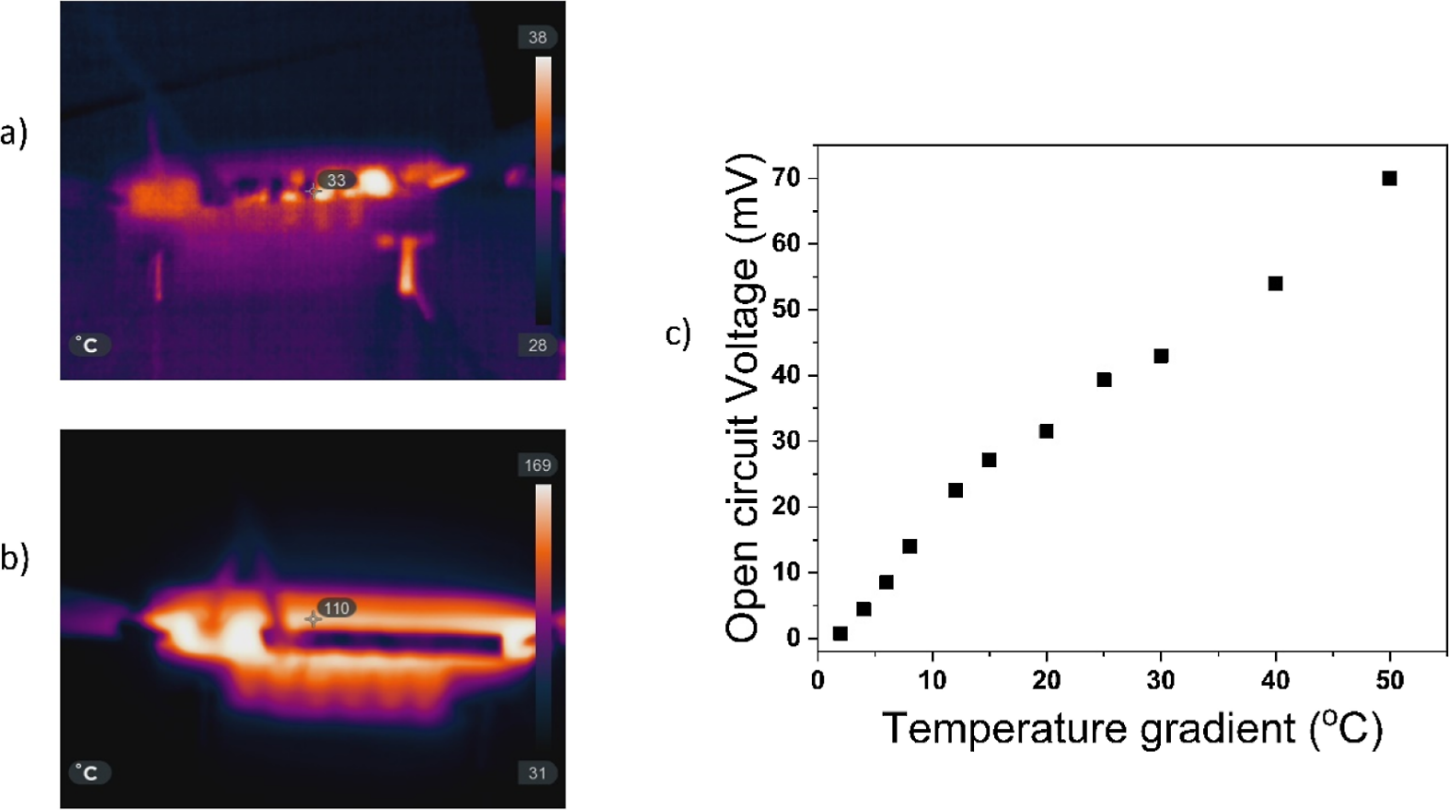
(a) Thermal image of the paint-based TEG at a low temperature, (b) thermal image of the paint-based TEG at a high temperature, and (c) open-circuit voltage versus temperature gradient of the paint-based TEG.

The paint-based TEG is made up of six TE legs and conductive silver paste, and each TE leg has a dimension of 12 × 4 × 0.1 mm^3^. Although the paint-based TEG is intended to be used for low-temperature energy harvesting, it can be used for high-temperature applications. This statement can be corroborated by looking at Figure 7a,b where the hot side of the TEG is placed on a hot plate, and the temperature of the hot plate is allowed to vary from 0 to 160 °C. The cold side is air-cooled, and a thermal camera is used to record the temperatures. Even at the maximum hot side temperature of 160 °C, the module was undamaged and still operational. On cooling, the module was visually inspected, its internal resistance was remeasured, and no significant changes were found. This shows that the module can be operated at higher temperatures repeatedly for a long period of time. Figure 7c shows the variation of the open-circuit voltage with the temperature gradient. As expected, the voltage is monotonically increasing as a function of the temperature gradient. At the highest temperature gradient of 50 °C, a maximum open-circuit voltage of 70 mV is obtained.

**Figure 8:**
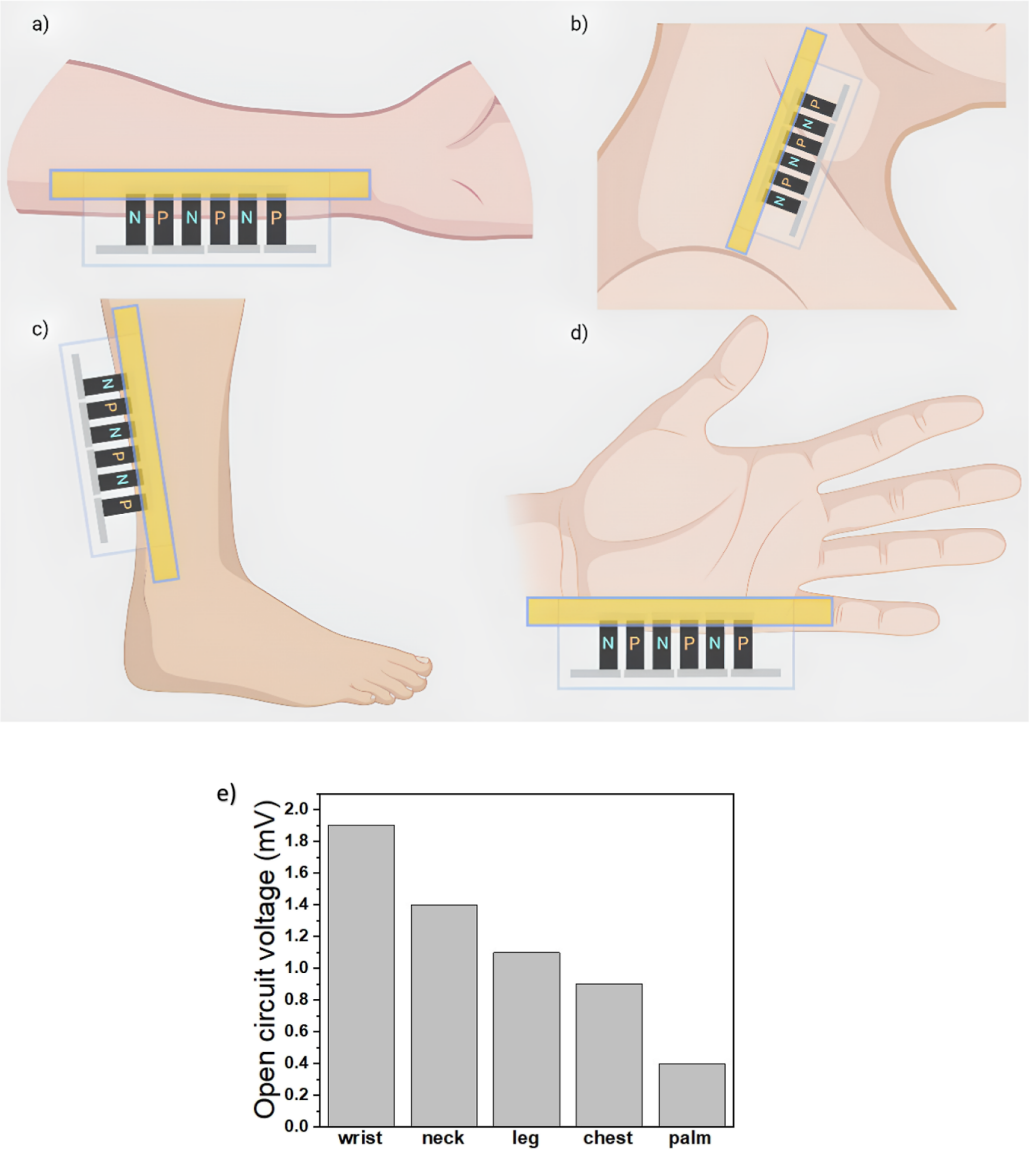
Application of the paint-based TEG: (a) wrist, (b) neck, (c) leg, and (d) palm and (e) open-circuit voltage.

Demonstration of the applications of the paint-based TEG can be seen in Figure 8: (a) hand, (b) neck, (c) leg, and (d) palm. In each case, the hot side is taped to the human body, while the cold side is suspended in the air. An open-circuit voltage is measured, as shown in Figure 8e; it can be seen that the hand (wrist) has the highest voltage of 1.9 mV, while the palm has the lowest voltage of 0.4 mV. These results indicate that the best position for wearable applications is the wrist. Furthermore, one important finding of this study is that higher performance is obtained when the cold side is suspended in the air, in comparison with when the cold side is in contact with the human body. For example, an open-circuit voltage of 1.5 mV is obtained when the TEG is worn on the wrist (both hot and cold sides are in contact with the body). The reason for the higher performance when the cold side is suspended in air is due to the increase in the air convection on the cold side, which led to the creation of a larger temperature gradient in comparison with the latter case. The performance is expected to increase by increasing the speed of air, for example, when the subject is walking/running. It should be noted that the measurements in Figure 8e were carried out in a laboratory at a room temperature of 28 °C; when the same measurements were repeated outdoors in the winter of Istanbul, Turkey, with an ambient temperature of 8 °C, the results increased by about fourfold. In conclusion, the paper-based TEG being lightweight and flexible can be worn comfortably by suspending the cold side in the air.

### 3.3 Performance Measurement of the Paint-Based TEG

**Figure 9:**
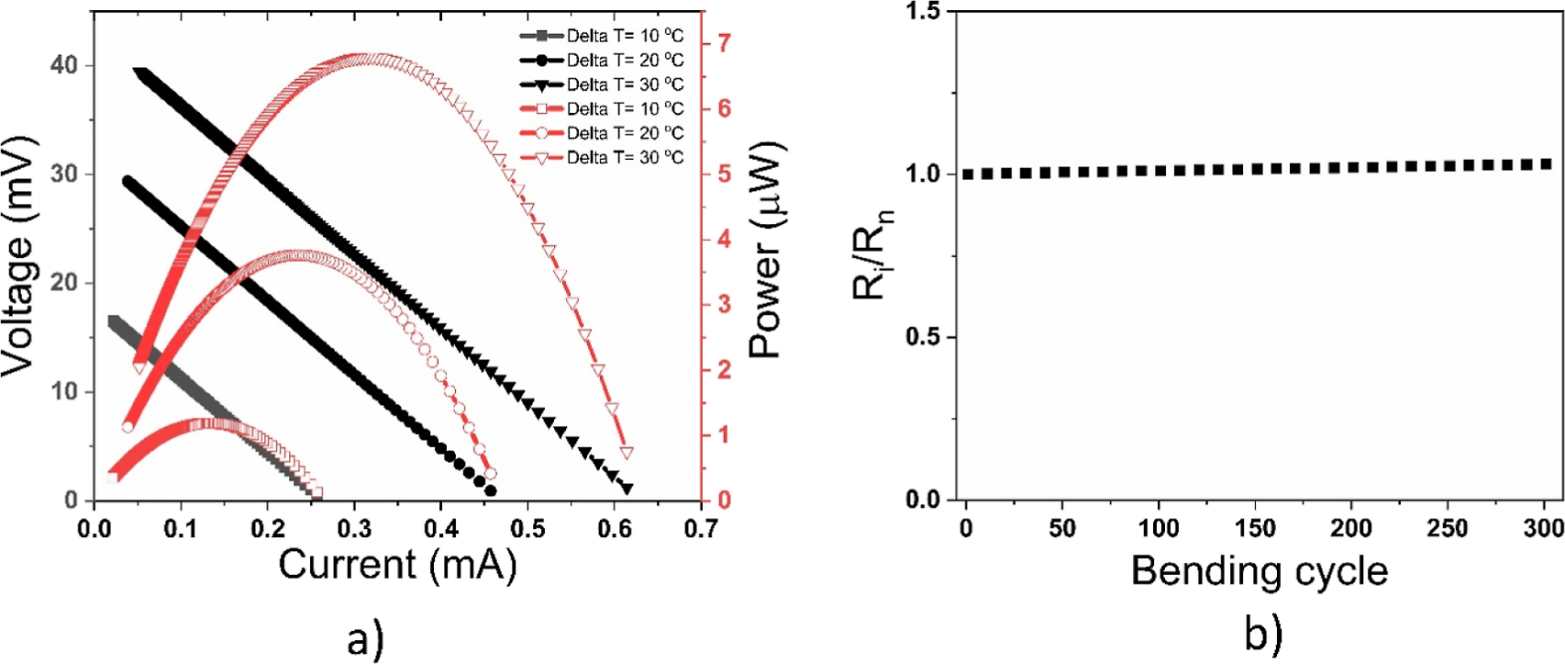
(a) Output performance of the paint-based TEG. (b) Variation of the internal resistance with bending cycle.

To further determine the performance of the paint-based TEG, voltage and current at different temperature gradients were measured to compute the output power as shown in Figure 9a. As expected, the output performance increases with an increase in the temperature gradient. At the temperature gradients of 10, 20, and 30 °C, the open-circuit voltages are 18.5, 32, and 42 mV, respectively. At the same temperature gradients, the corresponding short circuit currents are 0.257, 0.457, and 0.625 mA. The relationship between voltage and current is linear as given by eq 2, while that of the power and the current is nonlinear as given by eq 3. The output power is zero at open circuit and short circuit positions and have nonzero values otherwise. It is zero at an open circuit because the load current is zero; similarly, it is zero at a short circuit because the load resistance is zero. A maximum output power is said to be achieved when the electrical impedance matching is achieved as given by eq 4, herein, the maximum output powers are 1.19, 3.77, and 6.8 μW for the temperature gradients of 10, 20, and 30 °C, respectively. These results indicate that a reasonable amount of energy can be obtained from the paint-based TEG by increasing the temperature gradient. Similarly, the TE paint is cheap, nontoxic, highly reproducible, and can be easily applied to many surfaces, thereby pave the way to a large-scale conversion of heat into electrical energy.

A cycle bending test was carried out on the paint-based TEG as shown in Figure 9b. This is to determine the suitability of the paint-based TEG for wearable applications. The paint-based TEG is expected to undergo bending cycles, and that may lead to the variation of the internal resistance. If the internal resistance varies with the bending cycle, then the TEG would not be suitable for wearable applications. Herein, the ratio of the initial internal resistance and the internal resistance at the *n*th bending cycle is given in Figure 9b where the bending radius is 10 mm. It can be seen that variation of the internal resistance with the bending cycle is negligible even after 300 cycles. This shows that the paint-based TEG is flexible, durable, and mechanically stable for wearable applications.

Table 3 displays the performance comparison of paper-based TEGs, focusing on two key performance indicators: normalized open-circuit voltage (N_*V*_TE_) and normalized maximum output power (N_*P*_max_). Variations in TEG performance is primarily attributed to the materials and geometry of the TE legs. The data in Table 3 shows that TEGs using chalcogenides outperform others, with this study demonstrating N_*V*_TE_ and N_*P*_max_ values approximately 2.9 and 55 times higher than those in ref (39) respectively.

**Table 3:** Performance Comparison of Paper-Based TEGs

materials	pair	Δ*T* (K)	*V*_TE_ (mV)	N_*V*_TE_ [μV/(K pair)]	*P*_max_ (nW)	N_*P*_max_ [nW/(K pair)]
this work	3	30	42	466.67	6800	75.55
CuI and n-type Bi^40^	10	49	84.5	172	215	0.439
pencil traces and PEI-treated pencil traces^23^	5	60	9.2	30.67	1.75	0.0058
MWCNT and C-CNF^41^	2	70	3.3	23.57	3.7	0.0264
PEDOT:PSS, EMIM:TCM, and cellulose^42^	5	2.44	0.158	12.95		
Cu_2_SnS_3_ and n-type bismuth^39^	20	80	256	160	2180	1.36
Bi_2_Te_3_- and Sb_2_Te_3_-modified paper^43^	3	50	41.2	274.67		
Bi_2_Te_3_ and bacterial cellulose^44^	96	55	70.5	13.35	596	0.11

Chalcogenides contribute to high Seebeck coefficients and relatively low thermal conductivity near room temperature, while the addition of graphite enhances electrical conductivity. The geometry of the TE legs and interconnects is crucial, as longer legs create higher temperature gradients, resulting in increased open-circuit voltages. However, longer legs also have higher internal resistance, which can significantly reduce the output power. Wider legs can decrease internal resistance due to increased surface area, but there is a limit to how wide they can be as this would restrict the number of TE legs. Therefore, optimizing the geometry of paper-based TEGs is vital for achieving maximum performance.

## 4 Conclusions

A comprehensive study of an inexpensive, nontoxic, wearable, and highly reproducible paint-based TEG for heat energy harvesting is presented. The findings of this study can be summarized as follows.

- The optimum concentration of graphite (0.071 g), sodium silicate (0.13 mL), and TE compounds (0.429 g) were determined to obtain a higher performance of paint-based TE compounds.
- The highest power factors of the as-synthesized n- and p-type TE paints are 1.34 μW/(cm K^2^) and 1.42 μW/(cm K^2^), respectively, obtained at the temperature of 57 °C.
- The paint-based TEG can be subjected to a temperature of up to 160 °C without being damaged.
- For wearable applications, a higher output performance is obtained when the cold side of the TEG is allowed to float in the air in comparison to when it is in contact with the human body.
- When the performance of the paint-based TEG is measured on five parts of the body, namely, chest, palm, leg, wrist, and neck, the wrist has the highest open-circuit voltage of 1.9 mV, indicating the most suitable position for the wearable application.
- At temperature gradients of 10, 20, and 30 °C, the corresponding output power of 1.19, 3.77, and 6.8 μW is obtained, respectively.

## Data Availability

The data that support the findings of this study are available from the corresponding author upon reasonable request.
